# Supplementary material for: FabF and FadM cooperate to recycle fatty acids and rescue ∆plsX lethality in Staphylococcus aureus
Source: PLoS Genet. 2026 May 27;22(5):e1012165. doi: 10.1371/journal.pgen.1012165 (PMC13245860; doi:10.1371/journal.pgen.1012165)
Supplement: S1 Fig — Locus tags of genes related to this study are presented. Dark green, FASII initiation; blue, FASII elongation; black, acyl carrier protein (acpP); purple, phospholipid synthesis; olive, FASII bypass fatty acid kinase; pink, regulatory; orange, FadM; grey, not directly related. Locus tags are given for USA300 FPR_3797 and 8325–4 as references. (PDF) [file pgen.1012165.s001.pdf]

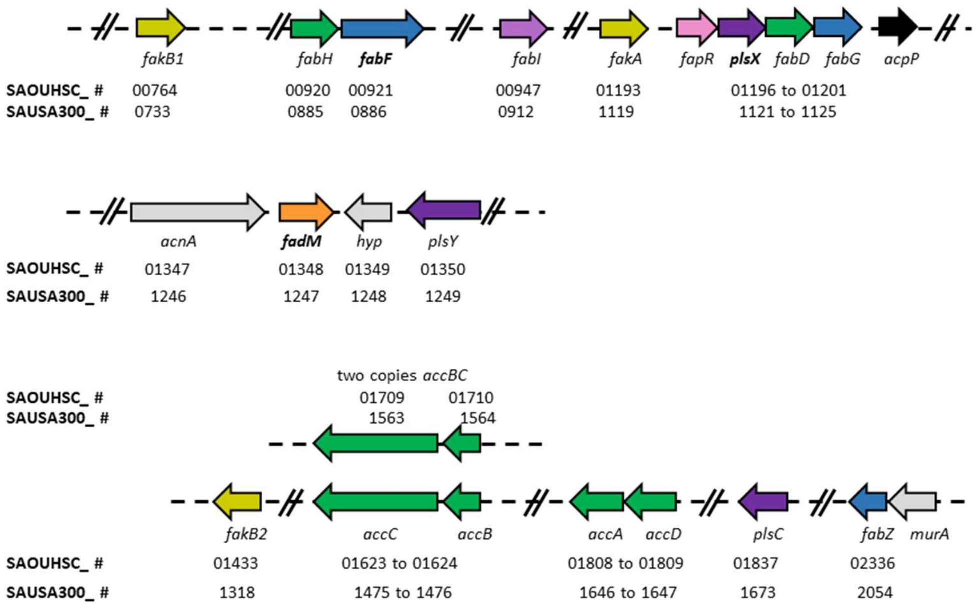

S1 Fig. FASII and phospholipid gene organization in RN-R and JE2 strains. Locus tags of genes related to this study are presented. Dark green, FASII initiation; blue, FASII elongation; black, acyl carrier protein (*acpP*); purple, phospholipid synthesis; olive, FASII bypass fatty acid kinase; pink, regulatory; orange, FadM; grey, not directly related. Locus tags are given for USA300 FPR_3797 and 8325-4 as references.
