# Supplementary material for: FabF and FadM cooperate to recycle fatty acids and rescue ∆plsX lethality in Staphylococcus aureus
Source: PLoS Genet. 2026 May 27;22(5):e1012165. doi: 10.1371/journal.pgen.1012165 (PMC13245860; doi:10.1371/journal.pgen.1012165)
Supplement: S2 Fig — RN-R ΔplsX strains containing an empty plasmid carrying a chloramphenicol resistance cassette (pIMAY [57]; left side of plates), or a plasmid carrying the plsX gene expressed from its native promoter on pIMAY (pPlsX; right side of plates), were streaked on BHI chloramphenicol (Cm) 10 µg/ml solid medium, without or with 250 µM C18:1 to bypass the ∆plsX defect. Background growth on BHI (left) may be due to FA carryover from pre-cultures or to FA traces in medium. Plates were photographed after 48h growth at 30°C. The ΔplsX strain carrying the empty plasmid failed to grow in the absence of C18:1 supplementation, while the pPlsX-complemented strain grew in both media. (PDF) [file pgen.1012165.s002.pdf]

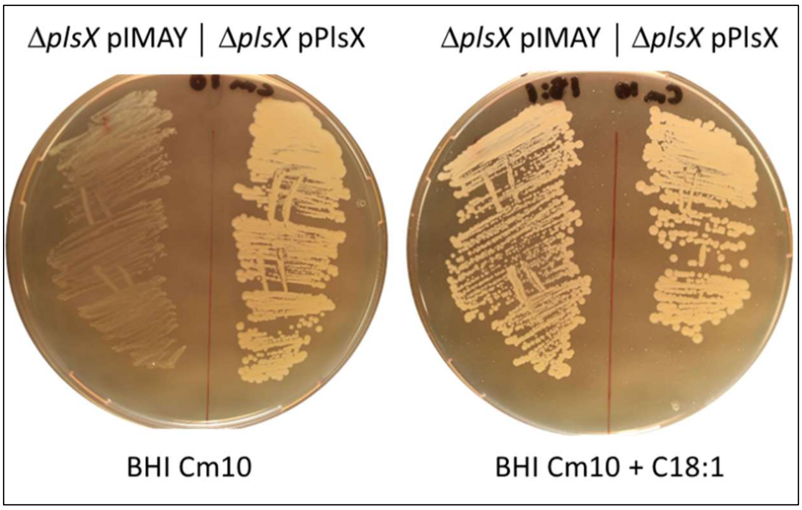

S2 Fig. Complementation of Δ*plsX* by a plasmid-carried *plsX* gene. RN-R Δ*plsX* strains containing an empty plasmid carrying a chloramphenicol resistance cassette (pIMAY [57]; left side of plates), or a plasmid carrying the *plsX* gene expressed from its native promoter on pIMAY (pPlsX; right side of plates), were streaked on BHI chloramphenicol (Cm) 10 µg/ml solid medium, without or with 250 µM C18:1 to bypass the Δ*plsX* defect. Background growth on BHI (left) may be due to FA carryover from pre-cultures or to FA traces in medium. Plates were photographed after 48h growth at 30°C. The Δ*plsX* strain carrying the empty plasmid failed to grow in the absence of C18:1 supplementation, while the pPlsX-complemented strain grew in both media.
