# Supplementary material for: FabF and FadM cooperate to recycle fatty acids and rescue ∆plsX lethality in Staphylococcus aureus
Source: PLoS Genet. 2026 May 27;22(5):e1012165. doi: 10.1371/journal.pgen.1012165 (PMC13245860; doi:10.1371/journal.pgen.1012165)
Supplement: S3 Fig — JE2 ∆plsX was grown overnight in BHI + C18:1. After washing, the strain was resuspended to OD600 = 0.05 and used to inoculate BHI medium to which platensimycin was added at the concentrations indicated below graphs. Left, growth curves with the different platensimycin concentrations added to medium. Right, the optimal concentration leading to improved growth with platensimycin (red line) compared to growth in BHI medium (black line). The averages of independent triplicates are shown. Growth kinetics of ΔplsX cultures in BHI and BHI supplemented with platensimycin (0.125 µg/mL) were analyzed across all time points using a mixed-effects model (two-way repeated measures; ANOVA using GraphPad Prism 10) with repeated measurements matched by subject. The model tests for effects of medium, time, and their interaction. A significant treatment effect was observed (p < 0.0001, ****). (PDF) [file pgen.1012165.s003.pdf]

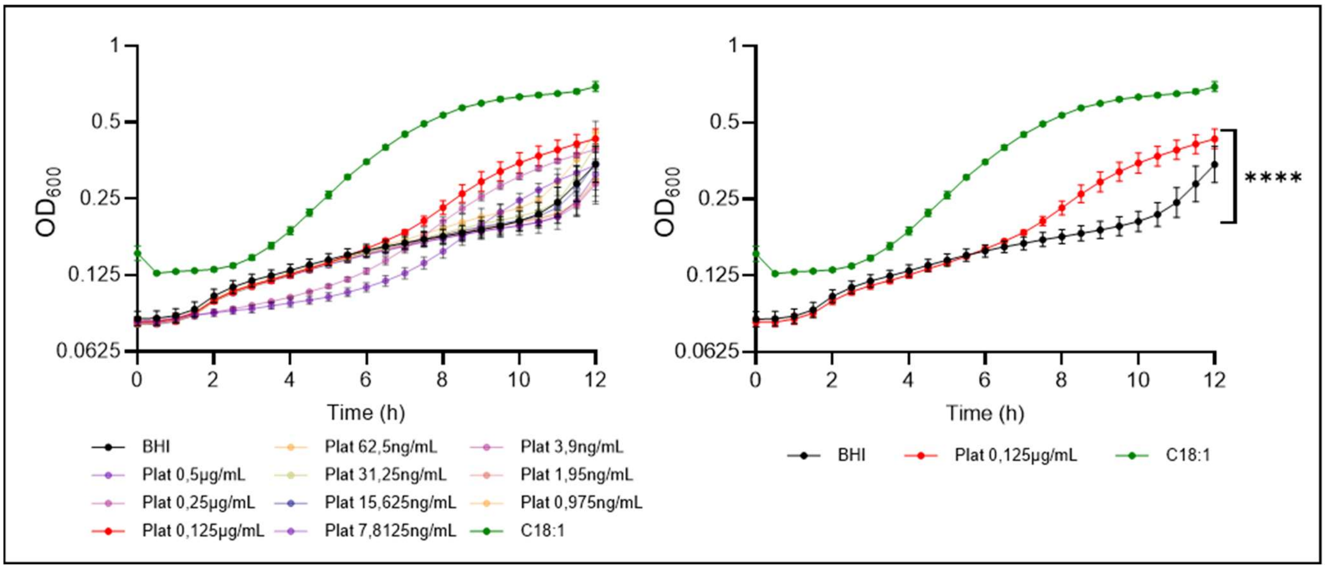

S3 Fig. JE2 Δ*plsX* growth stimulation by platensimycin in liquid culture. JE2 Δ*plsX* was grown overnight in BHI+C18:1. After washing, the strain was resuspended to OD_{600} = 0.05 and used to inoculate BHI medium to which platensimycin was added at the concentrations indicated below graphs. Left, growth curves with the different platensimycin concentrations added to medium. Right, the optimal concentration leading to improved growth with platensimycin (red line) compared to growth in BHI medium (black line). The averages of independent triplicates are shown. Growth kinetics of Δ*plsX* cultures in BHI and BHI supplemented with platensimycin (0.125 µg/mL) were analyzed across all time points using a mixed-effects model (two-way repeated measures; ANOVA using GraphPad Prism 10) with repeated measurements matched by subject. The model tests for effects of medium, time, and their interaction. A significant treatment effect was observed ($p < 0.0001$, ****).
