# Supplementary material for: FabF and FadM cooperate to recycle fatty acids and rescue ∆plsX lethality in Staphylococcus aureus
Source: PLoS Genet. 2026 May 27;22(5):e1012165. doi: 10.1371/journal.pgen.1012165 (PMC13245860; doi:10.1371/journal.pgen.1012165)
Supplement: S4 Fig — A. The fad operon encodes the acyl-CoA synthesis degradation pathway (Fad), including FadE, which converts FA to acyl-CoA, a FadM substrate [20]. Fad is subject to CcpA-glucose repression [25–28], so that acyl-CoA pools would drop in glucose-containing medium. Strains precultured in LB and LB with C18:1 for ΔplsX (Materials and Methods) were streaked directly on solid LB medium without or with 0.5% glucose. Results show that the FadMm suppressor phenotype is not subject to glucose repression, making a role for Fad unlikely. Background growth on LB (left) may be due to FA carryover from pre-cultures or FA traces in medium. Upper and lower rows, strains are derived from JE2 and from RN-R, respectively. Results represent biological triplicates. (PDF) [file pgen.1012165.s004.pdf]

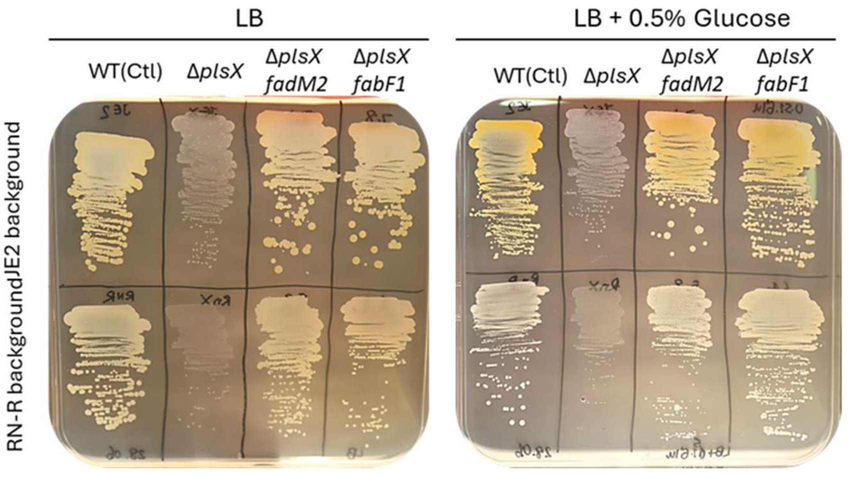

S4 Fig. FadM^{m} acyl-CoA thioesterase activity is not needed for Δ*plsX* suppression. A. The *fad* operon encodes the acyl-CoA synthesis degradation pathway (Fad), including FadE, which converts FA to acyl-CoA, a FadM substrate [20]. Fad is subject to CcpA-glucose repression [25-28], so that acyl-CoA pools would drop in glucose-containing medium. Strains precultured in LB and LB with C18:1 for Δ*plsX* (Materials and Methods) were streaked directly on solid LB medium without or with 0.5% glucose. Results show that the FadM^{m} suppressor phenotype is not subject to glucose repression, making a role for Fad unlikely. Background growth on LB (left) may be due to FA carryover from pre-cultures or FA traces in medium. Upper and lower rows, strains are derived from JE2 and from RN-R, respectively. Results represent biological triplicates.
