# Supplementary material for: FabF and FadM cooperate to recycle fatty acids and rescue ∆plsX lethality in Staphylococcus aureus
Source: PLoS Genet. 2026 May 27;22(5):e1012165. doi: 10.1371/journal.pgen.1012165 (PMC13245860; doi:10.1371/journal.pgen.1012165)
Supplement: S5 Fig — The indicated JE2 and ∆plsX derivative strains were grown overnight in BHI medium without (A) or with 250 µM C18:1 (B). Cultures were resuspended to OD600 = 0.1 and 100 µl were spread on the cognate solid media. Amoxicillin Etest strips were placed on plates, followed by 48 h incubation at 37°C. The amoxicillin MICs are indicated in micrograms per milliliter below each photo. (PDF) [file pgen.1012165.s005.pdf]

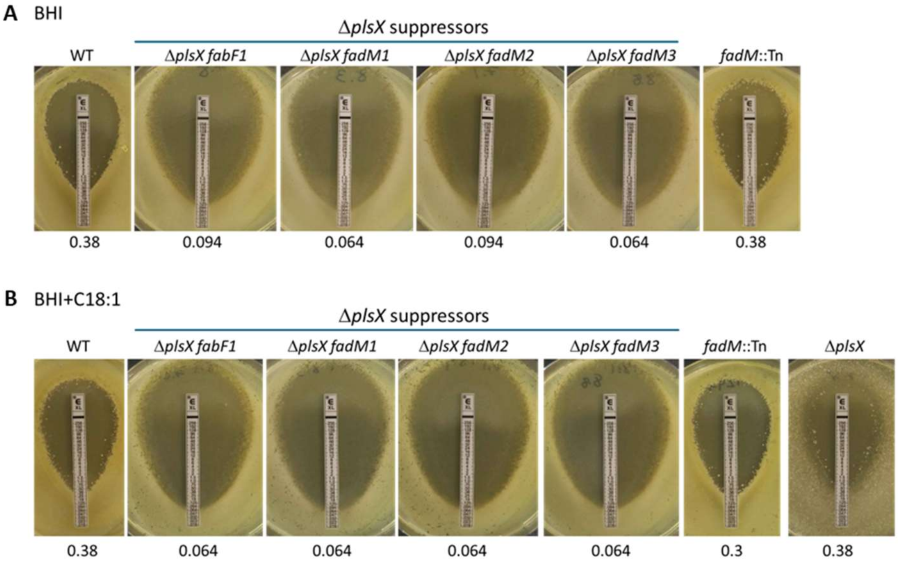

S5 Fig. MRSA JE2 Δ*plsX* suppressor mutants are sensitized to the β-lactam antibiotic amoxicillin. The indicated JE2 and Δ*plsX* derivative strains were grown overnight in BHI medium without (**A**) or with 250 µM C18:1 (**B**). Cultures were resuspended to OD_{600} = 0.1 and 100 µl were spread on the cognate solid media. Amoxicillin Etest strips were placed on plates, followed by 48 h incubation at 37°C. The amoxicillin MICs are indicated in micrograms per milliliter below each photo.
