# Supplementary material for: FabF and FadM cooperate to recycle fatty acids and rescue ∆plsX lethality in Staphylococcus aureus
Source: PLoS Genet. 2026 May 27;22(5):e1012165. doi: 10.1371/journal.pgen.1012165 (PMC13245860; doi:10.1371/journal.pgen.1012165)
Supplement: S6 Fig — Overnight BHI cultures of JE2 and fadM::Tn (SAUSA300_1247) were washed in 0.9% NaCl, pellets were resuspended to OD600 = 0.1, and 250 µl was spread on 120 x 120 mM square petri plates containing solid BHI medium. Three µg platensimycin were spotted on plates (‘P’ in white circle; N = 2). Plates were photographed at 48 h. Vertical arrow, inhibition zone; left arrow, growth ring surrounds the platensimycin deposit site in the WT, but not the fadM::Tn strain. (PDF) [file pgen.1012165.s006.pdf]

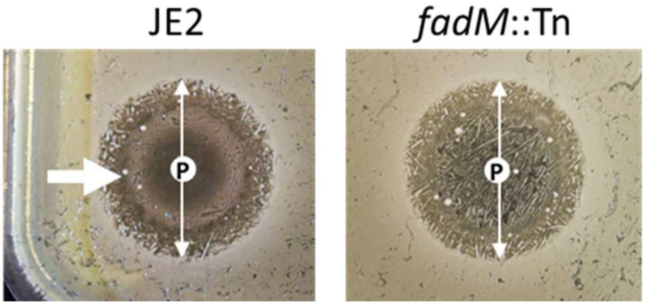

S6 Fig. FadM is required for platensimycin-stimulated ring formation in the WT background. Overnight BHI cultures of JE2 and *fadM::*Tn (SAUSA300_1247) were washed in 0.9% NaCl, pellets were resuspended to OD_{600} = 0.1, and 250 µl was spread on 120 x 120 mM square petri plates containing solid BHI medium. Three µg platensimycin were spotted on plates ('P' in white circle; N=2). Plates were photographed at 48 h. Vertical arrow, inhibition zone; left arrow, growth ring surrounds the platensimycin deposit site in the WT, but not the *fadM*::Tn strain.
