# Supplementary material for: FabF and FadM cooperate to recycle fatty acids and rescue ∆plsX lethality in Staphylococcus aureus
Source: PLoS Genet. 2026 May 27;22(5):e1012165. doi: 10.1371/journal.pgen.1012165 (PMC13245860; doi:10.1371/journal.pgen.1012165)
Supplement: S2 Table — (DOCX) [file pgen.1012165.s008.docx]

**S2 Table. Δ*plsX* suppressors ^a,b^.**

|  | **Gene : protein mutations in Δ*plsX* suppressor mutants** | | | | |
| --- | --- | --- | --- | --- | --- |
|  | SAUSA300_0886 FabF  (3-oxoacyl-ACP synthase II) | | SAUSA300_1247 FadM  (acyl-CoA thioesterase/ACP binding [20, 22-24]) | | |
| Strain name | *fabF1*: FabF^A119E^ | *fabF2*: FabF^D266A^ | *fadM1*: FadM^I38T^ | *fadM2*:  FadM^Y90F^ | *fadM3*: FadM^Y133F^ |
| RN4220 5.1 | X |  | NT | NT | NT |
| RN4220 5.2 | X |  | NT | NT | NT |
| RN4220 5.3 | X |  | NT | NT | NT |
| RN4220 5.4 | X |  | NT | NT | NT |
| RN4220 5.5 | X |  | NT | NT | NT |
| RN4220 5.6 |  |  |  | X |  |
| **RN4220 5.7** |  |  |  | **X** |  |
| RN4220 5.8 |  |  |  | X |  |
| RN4220 6.1 | X |  | NT | NT | NT |
| RN4220 6.2 | X |  | NT | NT | NT |
| RN4220 6.3 | X |  | NT | NT | NT |
| RN4220 6.4 | X |  | NT | NT | NT |
| RN4220 6.5 | X |  | NT | NT | NT |
| RN4220 6.6 | X |  | NT | NT | NT |
| RN4220 6.7 |  |  |  | X |  |
| RN4220 6.8 | X |  | NT | NT | NT |
| **JE2 7.1** |  |  |  | **X** |  |
| JE2 7.2 |  |  | X |  |  |
| JE2 7.3 |  |  |  | X |  |
| JE2 7.4 |  |  | X |  |  |
| **JE2 7.5** |  |  | X |  |  |
| JE2 7.7 | X |  | NT | NT | NT |
| JE2 7.8 | X |  | NT | NT | NT |
| JE2 8.1 |  |  | X |  |  |
| JE2 8.2 |  |  |  | X |  |
| JE2 8.3 |  |  | X |  |  |
| JE2 8.4 |  |  | X |  |  |
| JE2 8.5 |  |  | X |  |  |
| JE2 8.6 |  |  | X |  |  |
| JE2 8.7 |  |  |  |  | X |
| JE2 8.8 |  |  |  |  | X |
| **JE2 Sup1 ^b^** | X |  |  |  |  |
| **JE2 Sup2 ^b,c^** |  | X |  |  |  |

^a^ ‘X’ indicates that the mutation is present in the tested isolate. NT, not tested; without indication, no mutation detected by DNA sequencing. Suppressors identified from whole genome sequencing are in bold. ^b^ Sup1 and Sup2 suppressors of Δ*plsX* had the same secondary mutation in SAUSA300_100 (generating Csa1A^K185Q^), which was absent in subsequently isolated suppressors mapping to *fabF*. ^c^ Sup2, encoding FabF^D266A^, could not be propagated after storage.

^b^ Accompanying Excel S1 Table. Sequence comparisons of Δ*plsX* suppressors and reference strains.
