# Supplementary material for: FabF and FadM cooperate to recycle fatty acids and rescue ∆plsX lethality in Staphylococcus aureus
Source: PLoS Genet. 2026 May 27;22(5):e1012165. doi: 10.1371/journal.pgen.1012165 (PMC13245860; doi:10.1371/journal.pgen.1012165)
Supplement: S4 Table — (DOCX) [file pgen.1012165.s010.docx]

**S4 Table. Genetic materials.**

| **Strains** | | | |
| --- | --- | --- | --- |
| **Host strain** | **Strain** | **Description** | **Reference** |
| JE2 USA300_FPR3757 | JE2 (WT) | USA300_FPR3757 cured of its three native plasmids | [28] |
|  | Δ*plsX* | In-frame *plsX* deletion leaving operon intact | This study |
|  | Δ*plsX* suppressor in *fadM*; *ΔplsX fadM1* | *fadM1* encodes FadM^I38T^ | This study |
|  | Δ*plsX* suppressor in *fadM; ΔplsX fadM2* | *fadM2* encodes FadM^Y90F^ | This study |
|  | Δ*plsX* suppressor in *fadM; ΔplsX fadM3* | *fadM3* encodes FadM^Y133F^ | This study |
|  | Δ*plsX* suppressor in *fabF; ΔplsX fabF1* | *fabF1* encodes FabF^A119E^ | This study |
|  | *fadM* Tn insertion | JE2 derivative containing an erythromycin-resistance-marked transposon (Tn) insertion in SAUSA300_1247 at position 1384915 (based on JE2 genome entry NZ_CP020619) | [28] |
|  | Δ*plsX fadM::*Tn | Φ80 phage transduction of the SAUSA300_1247 Tn insertional mutant into the Δ*plsX fadM2* (7.1, **Table S2**) suppressor | This study |
| RN-R 4220 | RN-R 4220 (WT) | RN4220 derivative repaired for *fakB1* | [15] |
|  | Δ*plsX* | In-frame deletion of *plsX* leaving operon intact | This study |
|  | Δ*plsX* suppressor in *fadM; ΔplsX fadM2* | *fadM2* encodes FadM^Y90F^ | This study |
|  | Δ*plsX* suppressor in *fabF; ΔplsX fabF1* | *fabF1* encodes FabF^A119E^ | This study |
| **Plasmids** | | | |
|  | **Plasmid** | **Description** | **Reference** |
|  | pMAD | Thermosensitive plasmid used to generate double-crossover deletion events | [42, 43] |
|  | pMAD-*fapR-fabD* fragments | Used to generate *plsX* deletion | This study |
|  | pIMAY | Used for *plsX* cloning | [44, 57] |
|  | pIMAY-*plsX* | Used for *plsX* complementation in Δ*plsX* | This study |
| **Transducing phage** | | | |
|  | **Phage** | **Description** | **Reference** |
|  | φ80 | Used to transduce erythromycin-resistance-marked Tn insertion of SAUSA300_1247 into Δ*plsX fadM2* and Δ*plsX fabF1* backgrounds | [49] |
