# Supplementary material for: FabF and FadM cooperate to recycle fatty acids and rescue ∆plsX lethality in Staphylococcus aureus
Source: PLoS Genet. 2026 May 27;22(5):e1012165. doi: 10.1371/journal.pgen.1012165 (PMC13245860; doi:10.1371/journal.pgen.1012165)
Supplement: S5 Table — (DOCX) [file pgen.1012165.s011.docx]

**S5 Table. Oligonucleotides (5’ to 3’) used for genetic constructions and mutant detection**.

| **Construction type and primers used for detection** | | | **Description** |
| --- | --- | --- | --- |
| **Plasmid for Δ*plsX* construction on pMAD linearized by SmaI** | | |  |
| pMAD_FapR_F | TCTATCGATGCATGCCATGGTACCCCTTCATCACAGACCATGAACTAAGC | | *fapR* fragment |
| FapR_FabD_R | CGGAAAAATAATTGCTGTTTTACTCATAATTTTATCCTCGCTTATCATAAAAC | |  |
| FapR_FabD_F | GTTTTATGATAAGCGAGGATAAAATTATGAGTAAAACAGCAATTATTTTTCCG | | *fabD* fragment |
| FabD_pMAD_R | GCAGAAGCTTCTAGAATTCGAGCTCCCTGTGACCTGAAACAACAATTTGACC | |  |
| **Verifications for pMAD constructions** | | |  |
| pMAD_F | GTTACGTTACACATTAACTAGACAG | |  |
| pMAD_R | TGGACAGCATGGCCTGCAACGC | |  |
| **Cloning *plsX* on pIMAY-derived locus1 plasmid** | | |  |
| pIMAY_FapR_F | AGTTTGTGGTTTAATGGGGTTGACACTTGAACTATTTAAA | | *fapR* fragment |
| FapR_PlsX_R | TCAATTGCTAATTTAACCATTTTTTAGTACCTAGTCTTAA | |  |
| FapR_PlsX_F | TTAAGACTAGGTACTAAAAAATGGTTAAATTAGCAATTGA | | *plsX* fragment |
| plsX_pIMAY_R | TTGTTGCTATAATGAAATGCGTTCTTACTCATTTGATTCACCTA | |  |
| pIMAY_FapR_R | TTTAAATAGTTCAAGTGTCAACCCCATTAAACCACAAACT | | pIMAY linearized by reverse PCR |
| plsX_pIMAY_F | TAGGTGAATCAAATGAGTAAGAACGCATTTCATTATAGCAACAA | |  |
| **Δ*plsX* suppressor verifications** | | |  |
| FabF_F | GGAATCAGCTAGAGAACGC | | SAUSA300_0886 |
| FabF_R | CATTTTTCAACCCACAAGAGG | |  |
| 1247_F | AATCGGTATTTGAGGCAC | | SAUSA300_1247 |
| 1247_R | ATCTTCACGCTAATTACATT | |  |
| 0100_F | CGTGGCCTGGATATGATGAT | | SAUSA300_0100 *csa1* |
| 0100_R | GATTATCCTGAGCTACTTCG | |  |
| **Verifications of Tn insertion in SAUSA300_1247** | | | |
| 1247_F2 | GGATTACTATCGTCACGGTGG | Transposon insertion | |
| UpBursa_F | CTCGATTCTATTAACAAGGG |  |  |
| 1247_R2 | GTGTCCTGAACCTCGGAAGTTG |  | |
