# Supplementary material for: FabF and FadM cooperate to recycle fatty acids and rescue ∆plsX lethality in Staphylococcus aureus
Source: PLoS Genet. 2026 May 27;22(5):e1012165. doi: 10.1371/journal.pgen.1012165 (PMC13245860; doi:10.1371/journal.pgen.1012165)
Supplement: Attachment — Submitted filename: 26 Mar 7 Answers to Reviewers.docx [file pgen.1012165.s012.docx]

We thank Reviewers for their expert and thorough reading of this work. We did our best to address all questions and to clarify the text where points seemed unclear.

Reviewer #1:

The manuscript, entitled FabF and FadM cooperate to recycle fatty acids and rescue ΔplsX lethality in Staphylococcus aureus by Alexandra Gruss, describes the possible roles of FabF and FadM in phospholipid biosynthesis in S. aureus. I believe the pathway is significant in S. aureus envelope biogenesis, which involves both the membrane and teichoic acid. This study provides interesting insights into the flow of phospholipid biosynthesis in relation to the fatty acid biosynthesis system. However, the study heavily relies on phenotypic analysis alone, without proper genetic or biochemical assays, which should be addressed through extensive revision. I have several comments below.

Comments:
1. and 2.

1. Although there is some speculation about the effect of the FabF mutation (A119E) on FabF enzymatic activity, no clear evidence is presented to show whether the mutation has a positive or negative impact on the enzyme. The authors tried to introduce FabF (or the FabF mutant) into the wildtype background using a plasmid, but they were unable to obtain the desired mutants. As they mentioned, this may be due to toxicity from FabF overexpression. In that case, they should try this using an integrative plasmid under the control of the native promoter.

2. The authors used platensimycin to reduce the FAS system, which may be a good idea. However, the 96-hour culture was not a good choice, as it would likely yield unclear results due to the appearance of resistant mutants. Instead, they should test this in liquid culture while monitoring growth with a spike-in of the antibiotic.

To address Reviewer’s point about FabF(A119E) having positive or negative impact: Evidence for negative impact of the FabF A119E mutant on enzyme activity comes from results section (line 232) now entitled “Membrane FAs are shortened in *fabF* and *fadM* suppressors of Δ*plsX*.” and revised Fig. 4C, showing that FAs are overall shorter in Δ*plsX fabF1* compared to the WT strains in two strain backgrounds. The FabF mutant phenotype is compatible with a partially impaired acyl-ACP elongation defect as now explained in this section.

We failed to obtain the cloned *fabF* WT or mutant genes due to cloning difficulties, and we could not establish the *fabF1*-carrying plasmid into the Δ*plsX* mutant (paragraph line 147). Due to technical risks and resource limitations, we did not pursue this approach. Altogether, three results implicate FabF in Δ*plsX* suppression: 1- suppressors mapped to a single FabF point mutation, as isolated in two strain backgrounds, 2- the *fabF1* mutants display a shortened FA phenotype as expected if the protein is defective, and 3- the FabF inhibitor platensimycin leads to Δ*plsX* rescue (Fig. 2 and see below).

Reviewer suggested doing liquid assays to further test effects of platensimycin. While we did this, we note the following caveat: 1- Plate tests allow coverage of a broad range of platensimycin concentrations. The “ring” (e.g., Fig. 2A) indicates that the platensimycin concentration range stimulating growth is narrow. In liquid cultures, determining the optimal concentrations may vary according to conditions. 2- an inherent problem with liquid cultures is the outgrowth of both mutational suppressors and slow-growing platensimycin-adapted bacteria, making results potentially difficult to interpret.

For the suggested experiment (Supplementary Fig. S3), JE2 Δ*plsX* was grown in platensimycin concentrations ranging from ~8 ng/mL to 500 ng/mL in 2-fold increments. A significant but modest growth increase was observed when comparing growth in BHI and platensimycin optimally at 125 ng/mL (P=≤0.05 for comparisons between 7 and 12 h).

3. A similar issue applies to the FadM mutations. Although they showed the position of mutated residues in the structure, this remains speculative. No biochemical or direct genetic evidence was provided. Therefore, the effects of the FadM mutations on FadM function cannot be determined, and their interpretation is not valid based on speculation alone. The glucose test was indirect and insufficient. In this sense, the final sentence on page 6 (note: the manuscript lacks line numbers, which is very distracting) is not valid. To address this, they should test with a FadM thioesterase null mutant.

We apologize for having omitted line numbers that caused difficulties for reviewing. They are in the revised manuscript.

Crystal structure analyses and enzyme assays (Ref 20), and Alphafold modelling predictions, supported our suggestion that the FadM mutated residues all fall within the acyl binding cavity. We note that the three FadM suppressor mutations map within this restricted region that affects acyl thioesterase activity (new Ref 20). The revised text was modified to clearly state that this is speculation based on a previous study and Alphafold predictions.

As per Reviewer’s suggestion a FadM thioesterase null mutant was used as reference when examining FadM point mutations; see line 207 title “A *fadM* null mutant does not confer Δ*plsX* growth*‍*”. This control gave the first clear indication that at least some FadM activity is required to compensate the absence of PlsX. We then show that FadM is also required to obtain the *fabF* suppressors; line 294 section now entitled “FadM is required for emergence of Δ*plsX‍* suppressors mapping to FabF.”*.* Further support is given for this statement: the *fabF1* suppressor activity on Δ*plsX* is reversed when *fadM* is subsequently inactivated (Δ*plsX fabF1 fadM::*Tn requires exogenous FAs to grow: new Fig. 6B).

4. The experiment on page 8, regarding reverse PlsX activity, was very difficult to understand. The authors used a plsX knockout background, so where would the reverse activity come from? They seem to assume other salvage pathway that supplies FA-ACP from FA-PO4 ?, this experiment was quite confusing. They should explain this more clearly.

We thank Reviewer for pointing out the need to clarify the section on reverse PlsX activity. Reverse PlsX activity is used to convert free FAs (or exogenous FAs) that are first phosphorylated by Fak, from FA-PO_4_ to FA-ACP (illustrated in Fig. 1). To test whether Δ*plsX* suppressors *fabF1* or *fadM2* can also compensate the reverse PlsX activity., we used the fact that when FASII is blocked, *S. aureus* growth requires exogenous FAs and only reverse PlsX activity. We reasoned that if suppressors can also compensate this reverse activity, then they would also rescue growth when FASII is blocked and exogenous FAs are available. Our results showed that while suppressors restore PlsX ‘forward’ activity, they cannot compensate the PlsX ‘reverse’ activity. We hope that this is now clearer in the text and in Fig. 1 legend.

5. The final paragraph of the results section (page 8) claims that the absence of suppressors is evidence for a synthetic lethal relationship between FadM and FabF. However, simply the lack of suppressors alone is not direct evidence. The authors should show this with a FadM depletion assay or a relevant genetic approach.

Reviewer’s comment is partly answered in point 3. We showed no suppressors arise unless FadM is present (revised Fig. 6). We further support this conclusion by showing that the Δ*plsX* *fabF1* suppressor effect is lost when the *fadM::*Tn inactivated allele is introduced (Δ*plsX* *fabF1 fadM*::Tn) (Fig. 6B). Demonstration that *fabF* suppressors arise only if a *fadM* allele is intact strengthens the conclusion that FabF and FadM are functionally linked. We took care in the revised manuscript to refer to the possibility of FabF-FadM physical interactions as a working hypothesis.

Finally, we show that platensimycin-induced ring formation (mimicking FabF-mediated suppression) requires FadM even in the wild type background (new **Supplementary Fig. S6**).

****************************************************************************************

Reviewer #2:

In this manuscript, Dr. Wongdontree and colleagues investigate the mutations enabling the growth of plsX-deficient mutants on BHI agar plates. They uncover that genes fabF, fabM, and csa1a are mutated in ∆plsX suppressor strains. Furthermore, they demonstrate that platensimycin-mediated inhibition of FabF promotes the growth of plsX-deficient mutants on BHI agar plates without the need for fatty acid supplementation. Mechanistically, they propose an intriguing concept on how FadM and FabF cooperate to release endogenously produced fatty acids (FAs) from ACP to facilitate phospholipid synthesis in the absence of PlsX. Unfortunately, this model is supported by little to no genetic or biochemical evidence.

Major comments
1- It is unclear to the reader why this study was conducted in the first place. The results section starts with the construction of a plsX mutant without any explanation as to why. Given that such a mutant has already been previously described (DOI: 10.1111/mmi.12556), why did the authors consider it necessary to re-explore a seemingly well-characterized function of PlsX?

Why we constructed the mutant: The previously constructed *plsX* deletion strains in Ref 5 use RN4220 and an infrequently used strain, SA178R1 as genetic backgrounds. Although RN4220 is widely used, it lacks intact *fakB1* due to an internal deletion throughout the 8325 lineage (stated in Results). We therefore constructed the *plsX* deletion in an RN4220 strain in which *fakB1* was repaired, and in JE2 which carries an intact *fakB1* gene, and is commonly used by *S. aureus* researchers.

As to “why this study was conducted”: Our work on exogenous FA incorporation in *S. aureus* led us to investigate the step following FASII mediated by PlsX. We particularly wished to study the consequences of PlsX reverse activity in conditions where bacteria rely on exogenous FAs for growth. In the course of this construction, we noted the appearance of colonies on plates devoid of eFAs, which led us to the present study. In consideration of Reviewer’s questioning, we added the rationale in the Introduction.

2- Throughout this manuscript, the authors assume that BHI (brain heart infusion) broth/agar is devoid of fatty acids. This is misleading since BHI is a complex medium likely to contain FAs and other lipids. No doubt that FA supplementation rescues growth. However, no FA supplementation does not mean no FAs. The authors need to (i) either provide evidence that the BHI they used is devoid of FAs or (ii) thoroughly edit their manuscript to correct the assumption that environmental (exogenous) FAs are absent when S. aureus is grown in BHI.

We integrated Reviewer’s comment into the text: A 2023 publication reported the presence of C16:0 and C18:0 traces in BHI medium (Reference 16). BHI medium did not confer Δ*plsX* mutant growth, as Reviewer comments. The text was corrected as recommended (revised line 129).

3- I understand that FadM is required for the suppressor phenotype to be apparent in strains where mutations are located on FadM. However, for strains with FabF mutations, fadM::Tn transposon mutants are required to (i) show that FabF and FabM “cooperate” and (ii) FabF suppressors cannot grow without fatty acid supplementation in the absence of FadM.

The absence of any suppressors in the Δ*plsX* *fadM* null strain (neither in fadM nor in fabF) reasonably led us to conclude that FabF mutation emergence requires a functional FadM to be present, as presented. We agree however, that Reviewer’s suggested experiment strengthens this conclusion, who we thank for this suggestion.

As suggested the FadM::Tn allele was established in the Δ*plsX* *fabF1* suppressor strain. The resulting strain was able to grow in the presence of an exogenous FA source, but not on BHI plates, showing that *fadM* inactivation abolishes the *fabF1*-mediated suppression. This experiment, now presented in Fig. 6B indeed strengthens the conclusions.

We also added Supplementary Fig S6, in which the FabF inhibitor platensimycin was spotted on lawns of WT (JE2) and *fadM*::Tn strains. Both strains showed comparable inhibitory zones. However, a growth ring around platensimycin develops in the WT strain (24 h growth), but not in the *fadM*::Tn strain. These results give supporting evidence that FadM must be active to allow growth when FabF is partially disabled, here by antibiotic treatment.

4- The authors speculate that mutated FadM (FadMm) is better at binding ACP than wild-type FadM but provide no biochemical evidence of such an increased interaction. The same is true for decreased binding of FadMm to FAs.

We rewrote the statement, which was not meant as paraphrased by Reviewer. It is not that ‘FadMm is better at binding ACP’. Rather, we propose that the alterations in the FadM binding tunnel reduce FadM thioesterase activity, as was previously published for similar point mutants (Ref 20). Based on this, we hypothesize that the loss of one FadM activity would leave more FadMm available for ACP binding. Since the first submission, we applied Alphafold modelling to FadM and variants identified here and previously. The modelling predicts that acyl binding in the non-functional variants might be increased, not decreased in the FadM mutants, including those previously shown biochemically to have lost enzymatic activity (Ref 20). As this information is too preliminary to put forward, we modified the text to avoid overinterpretation of FadM mutant function, but propose in the working model that FadMm would be more available for ACP binding.

Minor comments

1- The reference genome used here (USA300_FPR3757 NC_007793.1) is odd since (i) USA300 JE2 complete genome is available on NCBI (CP020619.1) and (ii) RN-R parental strain is RN4220 whose whole genome has been sequenced previously (CP076105.1).

We used the most frequently used nomenclature to facilitate readers’ tasks of searching for additional genome information, and refer to the more commonly used USA300_FPR3757 and 8325 sequences to facilitate comparisons as used in most publications.

2- Phrasing is at times confusing throughout the manuscript. E.g., “PlsX forward, but not reverse activity”, “purified confirmed JE2 ΔplsX mutant”; “the primary stocked strain”; “disable the acyl-CoA binding cavity”; “phenotypes differed according to the parental strain”; “suppressor mutations do not compensate the PlsX reverse reaction”…

Thank you. The manuscript was reread and corrected for ambiguous and unclear wordings.

3- Related to minor comment 2: it is unclear to me what the authors mean with “a cloned fabF”. Is this mutagenesis? Is this overexpression on a (high or low copy number) plasmid? Is it with fabF own promotor? Have authors tried an inducible promotor?

We completed the description of cloning. For information, the native *fabF* and *fabF1* (renamed from *fabF^m^* by advice from Reviewer 3) genes were PCR-amplified onto a medium copy number vector and a constitutive promoter. We did not use an inducible promoter. Only the *fabF1* allele could be established in *E. coli* and in *S. aureus* RN-R and JE2 strains, but we failed to obtain either construct in the Δ*plsX* background.

4- Figure 2A: Plastenimycin is supposedly enabling the growth of Δplsx on BHI plates. The “growth ring” after 4 days highlighted by the authors is not obvious to me. What about growth assays in broth with increasing concentrations of plastenimycin? The ring after 6 days is more convincing (Fig 6 right).

The effects of platensimycin were confirmed in numerous repetitions of experiments, all giving the same results, although the times of incubation were varied. In Supplementary Fig. S6 a growth ring was observed after 24h in the WT strain (compared to *fadM*::Tn that has no ring).

Solid medium was used to distinguish the contributions of Δ*plsX* suppressor mutations, which give rise to colonies, from the stimulatory effects of platensimycin, which generates a homogenous growth ring. Both events might occur in a liquid culture.

We did perform experiments in liquid medium as suggested. JE2 Δ*plsX* was grown in platensimycin concentrations ranging from ~8 ng/mL to 500 ng/mL in 2-fold increments. A significant growth increase (P=≤0.05) for all points between 7 h and 12 h) was observed when comparing growth in BHI and platensimycin optimally added at 125 ng/mL. These supporting results are now presented in Supplementary Fig S3.

5- Relater to minor comment 4: the growth ring that is not “detected surrounding the platensimycin” (Fig. 6 left) is more obvious to me than the growth ring on Fig 2A left.

We are confused by this question: is Reviewer is referring to Fig. 2A right (not left) and Fig. 6 left? These differences may relate to variations in plate thickness or photo exposure, as experiments were done on different days. The essential findings are the same, i.e., that Δ*plsX* strain growth is stimulated by a narrow range of platensimycin concentrations.

6- Figure 4B. As a control experiment, acyl-ACP species for the strains all grown in BHI + C18:1 (not only JE2 ΔplsX) would be essential to draw any conclusion about effects specific to ΔplsX.

In Fig. 4B (now Fig. 4D), no part of the experiment was done in the presence of C18:1. This FA was only used in Δ*plsX* precultures, and then washed out by 2h FA starvation prior to sample preparation. Immunoblotting was performed in BHI medium for all samples. The supplementary acyl-ACP band in the Δ*plsX* strain cannot correspond to 18:1-ACP due to the absence of PlsX. Reviewer’s proposed experiment was published in reference 12, which is now discussed in the context of Fig. 4D (paragraph starting line 263).

7- Figure 5 left and Figure 5 right should read Figure 5A and Figure 5B, respectively.

Corrected.

8- Since SerFAs are present in all the strains (legend of Figure 5B), is it necessary to mention them for each strain? Also, JE2 should be replaced by WT.

Corrected as advised on Figure 5.

9- Figure 6: how does this look like without platensimycin? It could well be that fadM::Tn transposon mutants are more susceptible to platensimycin.

The effect of *fadM* inactivation independently of platensimycin addition is shown in revised **Fig. 6**. In addition, **Supplementary Fig. S6** shows that WT and *fadM*::Tn have equivalent inhibition zones around platensimycin. They differ by a growth ring that surrounds the platensimycin deposit position in the WT, but not the *fadM*::Tn strain.

**Reviewer #3**:

In the manuscript “FabF and FadM cooperate to recycle fatty acids and rescue plsX lethality in Staphylococcus aureus”, Wongdontree et al. report the isolation and characterization of strains harboring suppressor mutations that bypass the lethality of a plsX deletion in the absence of fatty acid supplementation. Since phospholipids are critical for cell viability, knowledge of the pathways and steps to synthesize phospholipids is important for the develop of antimicrobials that target membrane biogenesis. Thus, the study is highly significant and likely to be of broad interest to the scientific community. The authors use a series of genetics experiments and epistasis to characterize the mutations. The authors present evidence that suppressor alleles of fabF mimic the FASII inhibitor platensimycin and appear to be hypomorphic. FadM alleles encode proteins that retain FadM ACP binding activity but block acyl binding and thioesterase activity. Evidence is provided that the alleles cooperate and operate in the same pathway to provide suppression. The overall model proposed is that FadM facilitates ACP removal from the acyl-ACP-FabF and the variant FabF protein releases free fatty acids endogenously to restore membrane biogenesis, similar to exogenous FA addition. The FadM/FabF proteins cannot replace PlsX function in the reverse direction. Generally, the manuscript is well-written, and generally, conclusions are supported by the results. As a side note, no line numbers in the manuscript are provided. This makes it difficult to review. I always instruct my lab members to use line numbers to facilitate review. I did have a question as to how to refer to these alleles. FabF alleles seem hypomorphic, and FadM alleles seem to be function-specific, not gain-of-function alleles. My only major concerns, noted below, are related to results that imply certain conclusions, but a direct test is not performed. Additionally, there is no discussion. Even a short discussion section would increase the significance by relating how this is important perhaps clinically and clear up a lingering question about treating cells with AFN. Aside from these points, addressing a number of points below, referring to the text and figures will strengthen the manuscript and provide clarity.

We apologize for having omitted the line numbers and the difficulties incurred for reviewing.

We thank Reviewer for considering the term “gain-of-function mutations” that we used in the context of the mutations we described. Our intention was to assess the overall consequence of these mutations in providing a functional gain to bacteria, i.e., to grow in conditions under which they would not normally grow. However, we agree that both *fabF* and *fadM* mutant alleles led to concomitant loss of functions. We removed the term “gain-of-function mutations” in the revised text.

Major comments
• Page 6: “A positive role for acyl-CoA TE….” the experiment +/- glucose is indirect, and a negative result. Additionally, there are no reports yet showing Fad enzymes catalyze FA breakdown. Since the experiment does not yield useful information, I recommend it be removed. The following section “FadM inactivation…” is much more useful.

Recent reports show that Fad is active in processing exogenous FAs (References 25 and 26). We feel that the negative result showing that Fad gene repression does not affect mutational rescue helps rule out the need for one activity, leaving the hypothesis that FadM ACP-binding activity is involved in FabF rescue. We therefore prefer to keep this information in the text.

• Page 6: I agree that the result that the fadM::Tn mutation does not suppress is consistent with the notion that thioesterase activity is not needed but ACP binding is required, but this hasn’t been formally tested by making an catalytically inert active site substitution in FadM. Softening the language or emphasizing this point is needed. I feel that in Fig 3C the legend and model don’t quite align with the text. For instance in the legend the authors state that the point mutation reduces thioesterase activity.

We softened the language on P6-7 and Fig. 3C legend as Reviewer recommended, to propose a working model in which FadM acyl-CoA thioesterase activity is lower in the FadM point mutations. This is supported by crystallography results from Ref 20, as stated.

• In Fig 5, why is there a lag with JE2 SerFA treated with AFN? There is no discussion of this (no discussion section).

We added information on the lag associated with AFN-1252 treatment in the revised Introduction (line 101). This refers to our earlier studies showing an ~6-8 h time lag prior to FASII^bypass^ adaptation, described in Refs 6-8.

• The conclusions section ends abruptly. I understand that the authors wish to be succinct, but some sort of discussion would be helpful, especially in light of the point above. Also, I would have liked to have seen the authors tie this back to the biology. When would this be relevant? Do they assume that PlsX inhibitors could be developed, and the suppression needs to be considered?

We further developed the discussion to tie results with the biology of the system, as Reviewer recommends. We now discuss the fact that FadM is uncovered as a new factor involved in FASII and phospholipid homeostasis. Line 344: The relevance of the FabF-FadM connection is considered in ‘non-suppressor’ conditions: FadM may provide a ‘release valve’ that lowers FabF processivity to control FA chain length as may be needed in conditions of stress. Reviewer suggested that we discuss PlsX as a potential antimicrobial target: we prefer to not develop this subject, as it is our current research focus, and we prefer to avoid potential conflict for future patenting. In the course of revisions, we uncovered a new phenotype associated with suppressors, namely that both *fabF*- and *fadM*- Δ*plsX* suppressor strains are sensitized to β-lactam antibiotics as tested in an MRSA background (new supplementary Fig. S5). This more applied observation is discussed in the revised manuscript (Line 348).

Minor comments

• Fig 1 legend: check 4 lines from bottom – fill position 1 via PlsY, and position 2 by reverse PlsX activity and PlsC to produce PA?

Sentence corrected.

• Page 3, 3rd paragraph, first line: PlsX is dispensable in some bacteria?

This is explained (paragraph starting line 85) by giving examples, mainly related to either redundant functions or ACP thioesterases, which use FA-ACP as a substrate to generate FAs that feed into Fak.

• Page 4, 8th line: little? Do you mean incompletely characterized? Understudied? Please clarify.

We corrected the sentence and use the term ‘incompletely characterized’.

• Fig S1 legend: ORFs, not Orfs. Be consistent with abbreviations for 8325-4/SAOUHSC
• Fig S2 legend: plsX+ ORF with convention

Corrected.

• Page 5, line 5: fabFm? I don’t understand the allele designation. I suggest an allele number. If Sup1, how about fabF1? Same for fadMm2

We thank Reviewer, and corrected the designations as recommended, to *fabF1*, *fadM1*, *fadM2*, and *fadM3*.

• Page 5 “Attempts to demonstrate…” unclear what exactly the authors wish to do here. Do they mean to take the plsX deletion and express specifically either WT or the fabF mutant allele? Assessing dominant negative allele status? The section needs to be clarified. After continuing reading “As an alternative approach…” sounds like the authors wanted to ask whether disrupting FabF function was responsible for the suppression. Perhaps using this line as a transition will clarify. A hypomorphic allele of fabF that can be expressed in WT but not fabF+ allele is consistent with this.

We clarified the text and cloning strategy we used (Line 147—155), incorporated Reviewer’s transition sentence, and used the suggestion that the hypomorphic FabF allele is less toxic than the fully active WT allele, explaining its establishment in *S. aureus* JE2.

• Fig 2B: This is a working model. Please modify.

We now state that this, and also Fig. 7, are “working models” as suggested.

• Bottom of page 5: Do you mean DHNA-CoA thioesterase involved in menaquinone synthesis? Please clarify and define abbreviation 1,4-dihydroxy-2-naphthoyl-coenzyme A (DHNA-CoA)
• Page 6: “…main mutational suppressors” delete mutational. It’s redundant

Both comments are addressed and corrected.

• Page 6: “A positive role…” this is not very clear to me. Perhaps adding to an existing schematic how fatty acid synthesis and degradation occur and oppose one another would be helpful in visualizing this. Also, “If FadM acyl-CoA TE…” do you mean FadM acyl-CoA thioesterase activity? Please clarify and avoid abbreviations that obscure meaning to those who don’t think about these things every day. This will broaden interest. Also **unclear why reference 25 is used in this paragraph**. Same for Fig S3 legend. Finally, I recommend rephrasing the last sentence since the result suggest that the thioesterase activity is not responsible…

We rewrote the paragraph and removed confusing abbreviations. We also modified Fig. 1 to include an arrow showing that free fatty acids can be used by Fak or by fatty acid degradation (Fad) enzymes. The references 25 and 26 refer to studies reporting on Fad activity in *S. aureus*, which refutes a previous publication stating that this system was inactive in *S. aureus*. The last sentence in paragraph is corrected.

• NARSA – you mean Nebraska Transposon Mutant Library (NTML)? Please change.

Corrected as advised

• Page 6, “Suppression by fadM mutants…” When you say loss of FadM altogether, do you mean activity? Please clarify.

We corrected the phrasing to state ‘loss of all FadM activity’.

• Page 7: First paragraph – a better lead-in or rationale is needed for the membrane composition experiments

Line 232. The rationale sentence was added as recommended.

• Fig 4A: A stacked bar plot would more clearly show the changes in the proportions of membrane fatty acids.

We believe that the original FA profiles clearly illustrate the differences between strains, with shorter FAs in both suppressor backgrounds. The percent of long chain FAs C18:0 and C20:0, which are significantly decreased in both *fabF1* and *fadM2* suppressor backgrounds compared to WT, are now included in revised Fig. 4C.

• Fig 4B legend: for the acrylamide separation of acyl-ACPs, the reference provided is a review; it should be replaced with the report first showing the result. Should be replaced with Cronan, Jr 1982, JBC, which is cited in the review

We added the JBC 1982 article reference, and kept the review article, which explains the faster migration of acyl-ACP compared to free ACP in non-denaturing conditions.

• The “impact of plsX suppressors on bacterial growth” section seems out of place as positioned on page 7/8. I recommend moving this section earlier when the authors describe the isolation of the mutants.

Our intention was to group the characteristics of suppressors with respect to fatty acid metabolism and its consequences on growth. Based on Reviewer’s recommendation, we moved up the growth section, so that it precedes FA profiles (now Fig. 4A).

Also, in Fig 4C, the y-axes of the growth curves appears linear. This should be changed to a log10 or log2 scale due to exponential growth behavior of bacterial cells. Next, check the 4C panels (and throughout) – the curve legends say fadM and fabM. They should say fadM. Please change.

In Fig. 4C (now 4A), the Y-axis was already in a log2 scale. FadM is corrected in all legends.

Finally, the legend for Fig 4 is confusing because it mixes alleles and protein variants and the panels are the cryptic allele designations. I recommend using numerical alleles (as mentioned above) and putting the protein variants in parentheses in the graphs.

We homogenized Fig. 4 legend presentation by referring to the strain genotype with the encoded protein variants in parentheses, for example, “RN-R WT, and derivatives: Δ*plsX*, and Δ*plsX* suppressors *fabF1* (encoding FabF^A119E^) and *fadM2* (encoding FadM^Y90F^)”.

• Page 7: for acyl-ACP species variation section, variation should be vary. Also hybridization: do you mean probed since this is a western? Please clarify.

We corrected to “vary”, and changed “hybridization with” to “probing” as recommended.

• Page 8: when referencing Fig 5 and the top of the page, when Fig 5. Left, please change to Fig 5A. Same with Fig. 5 right (change to Fig 5B)

Corrected.

• Page 8, what is SerFA? Please define. Update: I found it in the methods. Please be sure to define in the text in the first instance as well as the background.

The SerFA medium is now defined on at its first mention (line 284).

• Page 8, For me, referencing Fig 1 is more helpful in thinking about the experiment about complementing reverse activity of PlsX. Recommend referencing Fig 1 and Fig 5A here.

Done as advised.

• Page 8-9, conclusions: I’m not sure these are officially gain-of-function mutations. Since they lose some function, maybe these would be better referred to as function-specific alleles?

The text in conclusions was modified in the sense of Reviewer’s correct comments, omitting the term gain-of-function. We did want to emphasize that although the mutation in FabF leads to lower activity, and those in FadM restrict its function, the net result is a survival gain for the bacterium. This is reformulated in Discussion section.

• Fig 7 is still a working model. Please change the title of this figure to reflect this. Overall working model? I find it hard to follow as drawn.

Fig. 7 title now reads “Proposed role of FabF - FadM interactions in controlling FA release and recycling from FabF intermediates”, which we believe implies tentativeness. We added the following statement to be clear on this: Our study showed epistasis between FabF and FadM, and leads us to speculate on how these proteins interact (depicted in the inset). We corrected the model in the inset for clarity.

• Materials and methods on page 10: please define LB as lysogeny broth and reference Bertani 1951 J Bacteriol

Done.

• Please provide some description of the method of determining fatty acid profiles as well as the reference provided. This makes it easier for readers to evaluate the method.

The revised manuscript is amended with the method and an original reference (ref 46).

**Have all data underlying the figures and results presented in the manuscript been provided?**
Large-scale datasets should be made available via a public repository as described in the *PLOS Genetics* [data availability policy](http://track.editorialmanager.com/CL0/http:%2F%2Fjournals.plos.org%2Fplosgenetics%2Fs%2Fdata-availability/1/010f0196302729ea-2ce45913-b87f-4f68-b994-11e561e66bd8-000000/UosiX0RazohCa_DJQ75hGkgLXIOvlVwBaO_DQS7hsxk=206), and numerical data that underlies graphs or summary statistics should be provided in spreadsheet form as supporting information.

Reviewer #1: Yes

Reviewer #2: **No:** Whole genome sequences of the suppressor mutants were not made available.

The entries are now entered in the Mendeley database: 10.17632/mzw2429w3m.1 as now stated in Materials and Methods section.

Reviewer #3: Yes
