# Supplementary material for: FabF and FadM cooperate to recycle fatty acids and rescue ∆plsX lethality in Staphylococcus aureus
Source: PLoS Genet. 2026 May 27;22(5):e1012165. doi: 10.1371/journal.pgen.1012165 (PMC13245860; doi:10.1371/journal.pgen.1012165)
Supplement: Attachment — Submitted filename: 26 Apr 29 Response to Reviewers.docx [file pgen.1012165.s013.docx]

We thank Reviewer 3 for the deep re-reading of our work. Please find below our responses to the remaining questions.

**Reviewer #3**:

The authors have done a nice job to address my previous comments. Thank you. I do wonder at Lines 249-261 why the 18:1 supplementation doesn’t change the susceptibility to b-lactams - wouldn’t that change the microdomain composition and Pbp2A’ oligomerization and activity? Perhaps it's too preliminary or outside the scope of the manuscript, but perhaps the authors can answer this question in the results section? Otherwise, I provide some suggestions for improving clarity.

We thank Reviewer for this question. We suggest two explanations for the β-lactam sensitivity of suppressors irrespective of FA in the medium. In Results, we write: “It was initially perplexing that FA addition did not restore amoxicillin resistance to levels of the Δ*plsX* mutant. One might speculate that *fabF* or *fadM* suppressor mutations induce stress, e.g., due to increased intracellular FAs, leading to expression changes affecting PBP2A’ folding or stability; alternatively, FA incorporation could be altered in suppressor strains. The mechanisms responsible for this phenotype remain to be elucidated.” Explaining methicillin-susceptibility of the suppressor strains when FA is added goes beyond the scope of this study, as Reviewer also noted.

• Lines 69-70: Technically isn’t PlsX and PlsC the first and third steps, with PlsY the second step? Please clarify in the text.

To clarify, we now write: “FabF for FA elongation *via* FASII, and PlsX/PlsY and PlsC for respectively the first and second steps of phospholipid synthesis (**Fig. 1**).”

• Fig 2 legend – for the dashed arrow – should be (right) plate.

Corrected. Thank you.

• Fig S3 – bilateral T test used for significance at all points between 7 and 12 h - is this the best way to compare?

We tried a few different tests to determine significance, and now show results across all time points using a mixed-effects model (two-way repeated measures; ANOVA). This analysis also shows a significant treatment effect (p<0.0001).

• Line 221: “The initial plsX deletion strains failed to grow…” Looking at Fig 4A, they do grow, but do not achieve an OD600 beyond ~0.16. This could be due to many things – depletion of the supplemented FAs from membranes. So “growth” is just residual until they run out of the nutrient. I recommend phrasing the phenotype less about growth and more about not getting past a specific population density.

We changed “fail to grow” to “show only residual growth, likely due to trace amounts of FA in medium (16)” as advised by Reviewer.

• Line 228-230: I feel like the conclusion here is vague…Do you mean that you expected more staphyloxanthin in the suppressor mutants? Please clarify here.

Yes, we thought that staphyloxanthin production in the USA300 background would improve suppressor growth compared to that in RN-R. However, all suppressors were far less pigmented than the parental strain (Fig. 4B). We clarified the text to write: “…However, decreased pigmentation of Δ*plsX*, and both *fabF* and *fadM* suppressors compared to WT and *fadM::*Tn controls (**Fig. 4B**) goes against this hypothesis. Possibly, stress response functions produced in JE2 but not in the σ^B^-defective RN-R strain [30] contribute to better growth of suppressors in the JE2 background.

• Line 245: “compared to those in the respective parental strains…and a concomitant increase in branched-chain fatty acids”? Please confirm this is what you mean, and complete the thought.

We corrected the sentence to write “compared to those in the respective parental strains…and a concomitant increase in shorter chain fatty acids”, as proportions of C16:0 are also increased.

• beta-lactam "sensitivity" - I believe the correct term is "susceptibility" to underscore that they are more vulnerable to the antibiotic.

Thank you for pointing out this subtility. We corrected the text.
